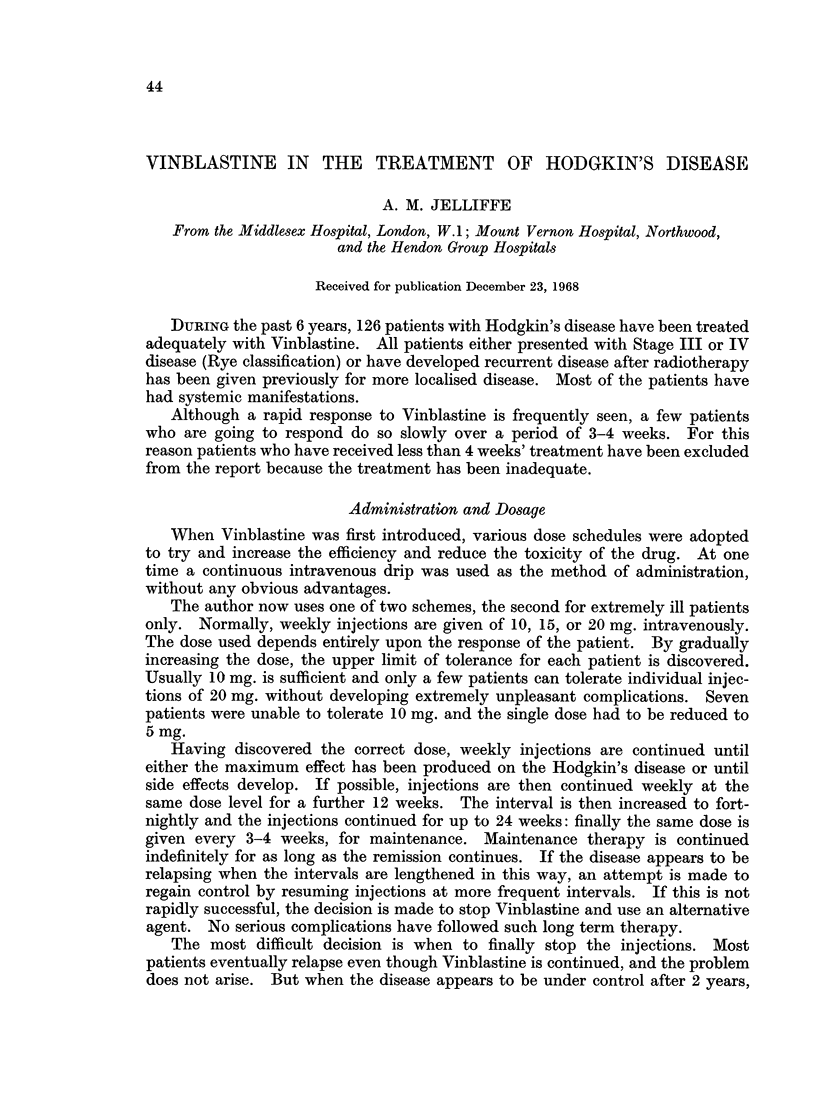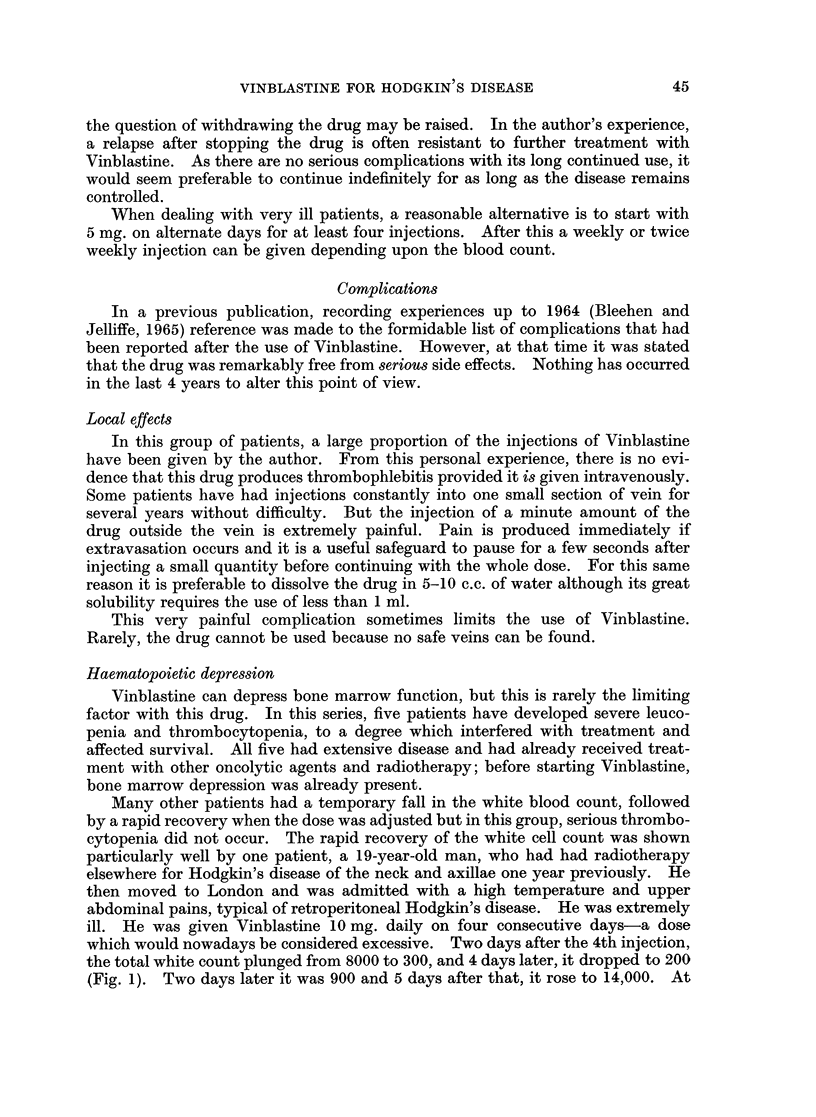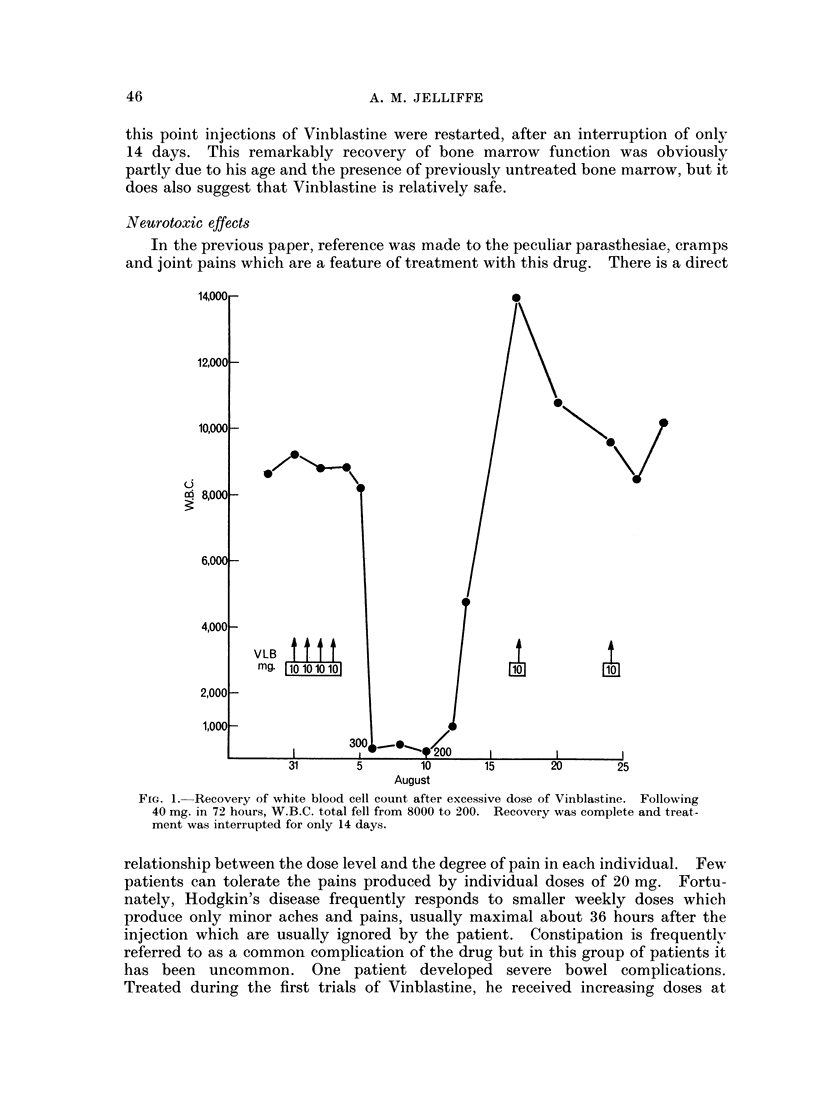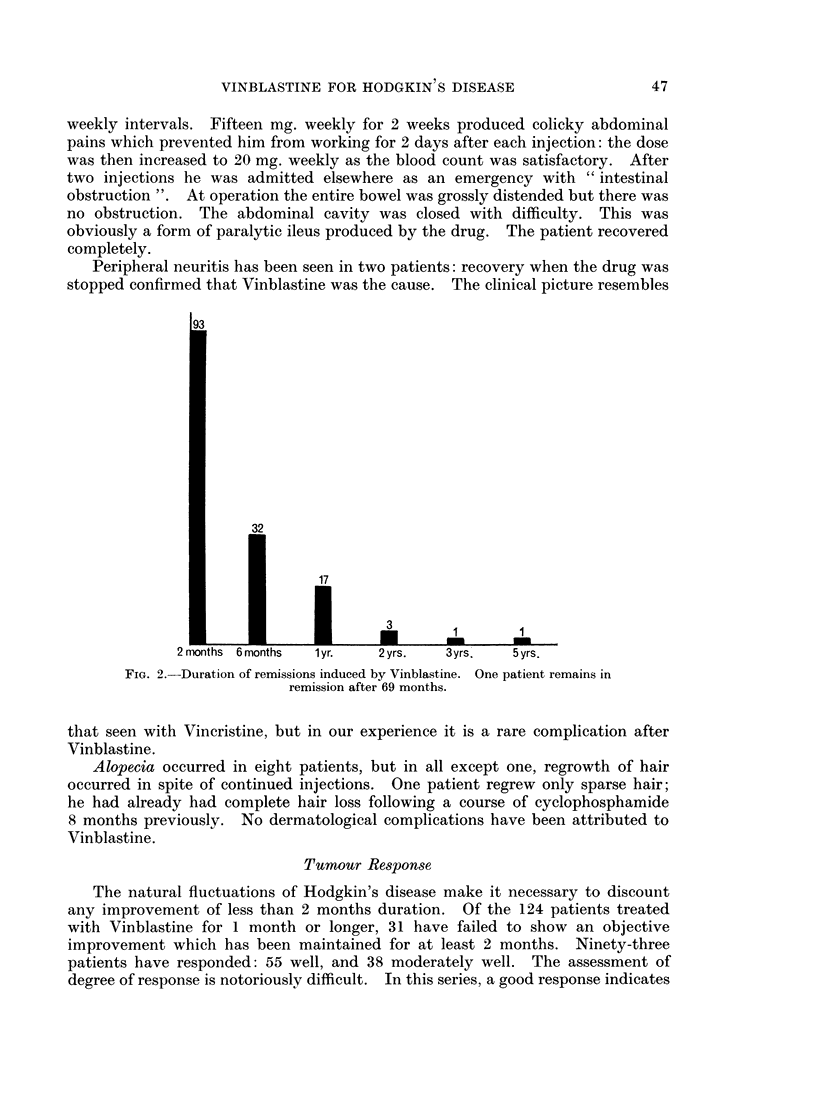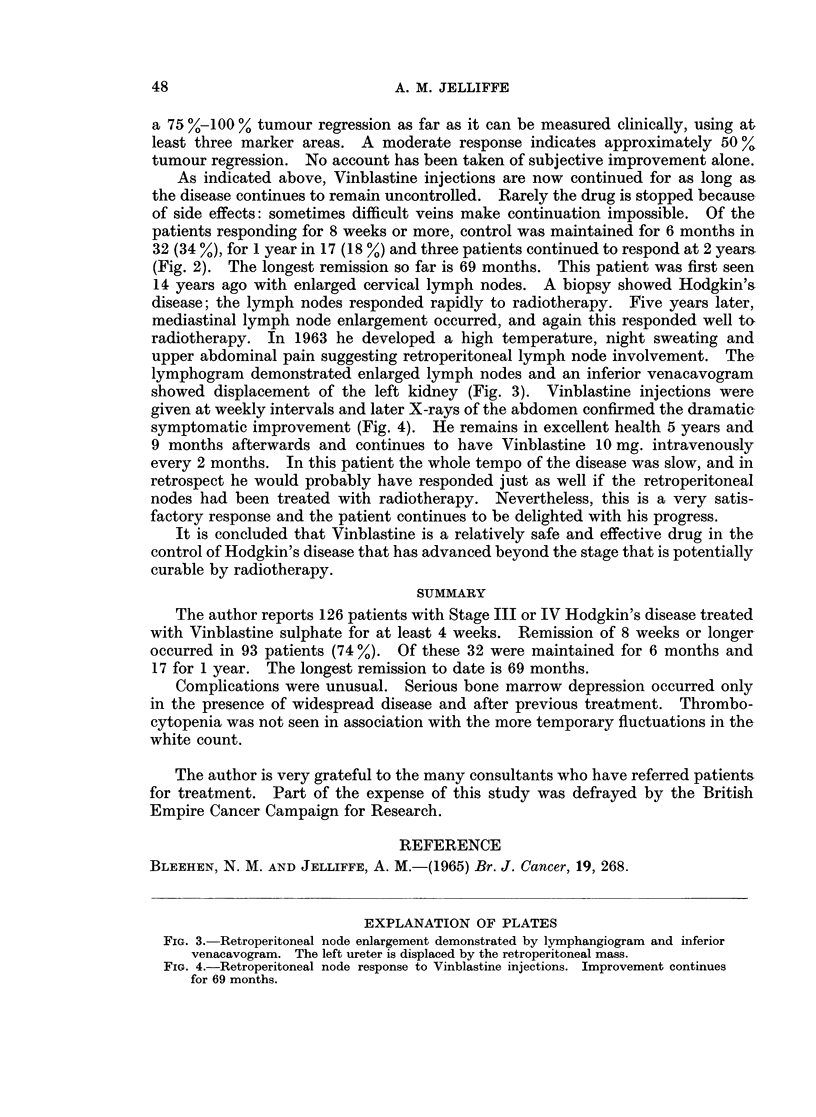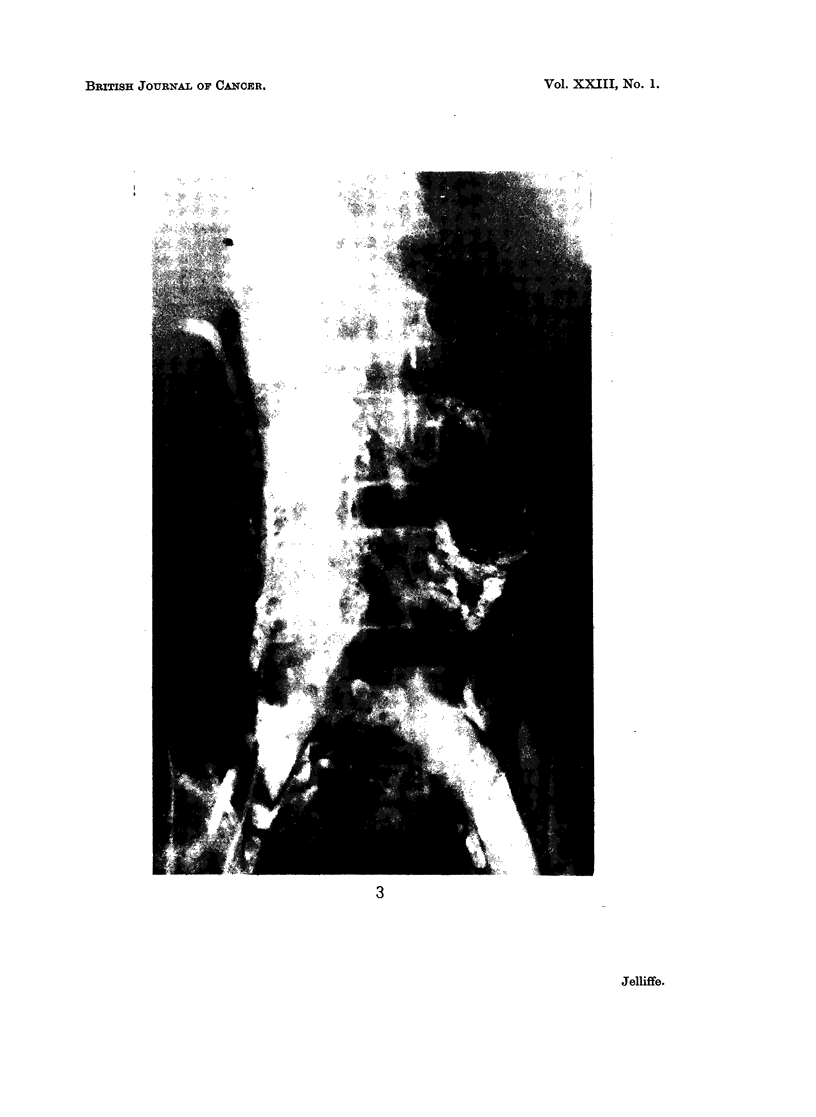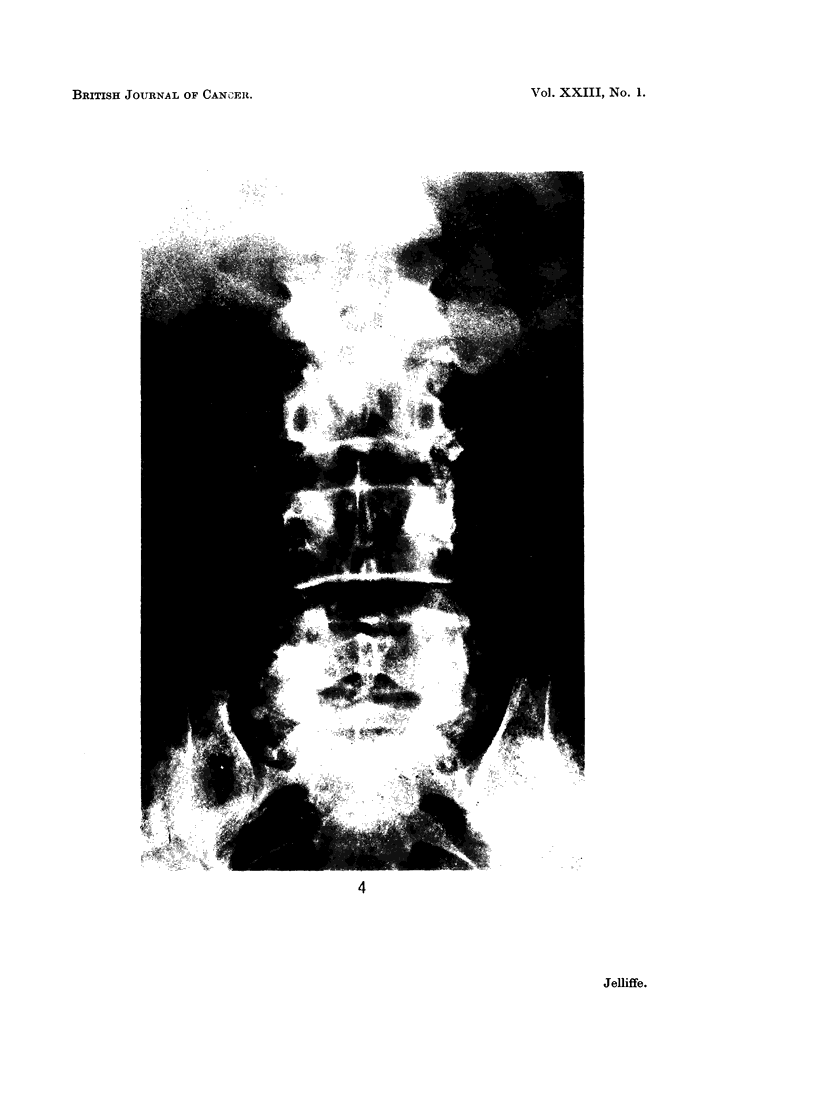# Vinblastine in the treatment of Hodgkin's disease.

**DOI:** 10.1038/bjc.1969.7

**Published:** 1969-03

**Authors:** A. M. Jelliffe

## Abstract

**Images:**


### VINBLASTINE IN THE TREATMENT OF HODGKIN'S DISEASE

A. M. JELLIFFE

*From the Middlesex Hospital, London, W.1; Mount Vernon Hospital, Northwood, and the Hendon Group Hospitals*

Received for publication December 23, 1968

DURING the past 6 years, 126 patients with Hodgkin's disease have been treated adequately with Vinblastine. All patients either presented with Stage III or IV disease (Rye classification) or have developed recurrent disease after radiotherapy has been given previously for more localised disease. Most of the patients have had systemic manifestations.

Although a rapid response to Vinblastine is frequently seen, a few patients who are going to respond do so slowly over a period of 3–4 weeks. For this reason patients who have received less than 4 weeks' treatment have been excluded from the report because the treatment has been inadequate.

### *Administration and Dosage*

When Vinblastine was first introduced, various dose schedules were adopted to try and increase the efficiency and reduce the toxicity of the drug. At one time a continuous intravenous drip was used as the method of administration, without any obvious advantages.

The author now uses one of two schemes, the second for extremely ill patients only. Normally, weekly injections are given of 10, 15, or 20 mg. intravenously. The dose used depends entirely upon the response of the patient. By gradually increasing the dose, the upper limit of tolerance for each patient is discovered. Usually 10 mg. is sufficient and only a few patients can tolerate individual injections of 20 mg. without developing extremely unpleasant complications. Seven patients were unable to tolerate 10 mg. and the single dose had to be reduced to 5 mg.

Having discovered the correct dose, weekly injections are continued until either the maximum effect has been produced on the Hodgkin's disease or until side effects develop. If possible, injections are then continued weekly at the same dose level for a further 12 weeks. The interval is then increased to fortnightly and the injections continued for up to 24 weeks: finally the same dose is given every 3–4 weeks, for maintenance. Maintenance therapy is continued indefinitely for as long as the remission continues. If the disease appears to be relapsing when the intervals are lengthened in this way, an attempt is made to regain control by resuming injections at more frequent intervals. If this is not rapidly successful, the decision is made to stop Vinblastine and use an alternative agent. No serious complications have followed such long term therapy.

The most difficult decision is when to finally stop the injections. Most patients eventually relapse even though Vinblastine is continued, and the problem does not arise. But when the disease appears to be under control after 2 years, the question of withdrawing the drug may be raised. In the author's experience, a relapse after stopping the drug is often resistant to further treatment with Vinblastine. As there are no serious complications with its long continued use, it would seem preferable to continue indefinitely for as long as the disease remains controlled.

When dealing with very ill patients, a reasonable alternative is to start with 5 mg. on alternate days for at least four injections. After this a weekly or twice weekly injection can be given depending upon the blood count.

### *Complications*

In a previous publication, recording experiences up to 1964 (Bleehen and Jelliffe, 1965) reference was made to the formidable list of complications that had been reported after the use of Vinblastine. However, at that time it was stated that the drug was remarkably free from *serious* side effects. Nothing has occurred in the last 4 years to alter this point of view.

#### *Local effects*

In this group of patients, a large proportion of the injections of Vinblastine have been given by the author. From this personal experience, there is no evidence that this drug produces thrombophlebitis provided it *is* given intravenously. Some patients have had injections constantly into one small section of vein for several years without difficulty. But the injection of a minute amount of the drug outside the vein is extremely painful. Pain is produced immediately if extravasation occurs and it is a useful safeguard to pause for a few seconds after injecting a small quantity before continuing with the whole dose. For this same reason it is preferable to dissolve the drug in 5–10 c.c. of water although its great solubility requires the use of less than 1 ml.

This very painful complication sometimes limits the use of Vinblastine. Rarely, the drug cannot be used because no safe veins can be found.

#### *Haematopoietic depression*

Vinblastine can depress bone marrow function, but this is rarely the limiting factor with this drug. In this series, five patients have developed severe leucopenia and thrombocytopenia, to a degree which interfered with treatment and affected survival. All five had extensive disease and had already received treatment with other oncolytic agents and radiotherapy; before starting Vinblastine, bone marrow depression was already present.

Many other patients had a temporary fall in the white blood count, followed by a rapid recovery when the dose was adjusted but in this group, serious thrombocytopenia did not occur. The rapid recovery of the white cell count was shown particularly well by one patient, a 19-year-old man, who had had radiotherapy elsewhere for Hodgkin's disease of the neck and axillae one year previously. He then moved to London and was admitted with a high temperature and upper abdominal pains, typical of retroperitoneal Hodgkin's disease. He was extremely ill. He was given Vinblastine 10 mg. daily on four consecutive days—a dose which would nowadays be considered excessive. Two days after the 4th injection, the total white count plunged from 8000 to 300, and 4 days later, it dropped to 200 (Fig. 1). Two days later it was 900 and 5 days after that, it rose to 14,000. At this point injections of Vinblastine were restarted, after an interruption of only 14 days. This remarkably recovery of bone marrow function was obviously partly due to his age and the presence of previously untreated bone marrow, but it does also suggest that Vinblastine is relatively safe.

*Neurotoxic effects*

In the previous paper, reference was made to the peculiar parasthesiae, cramps and joint pains which are a feature of treatment with this drug. There is a direct

Fig. 1.—Recovery of white blood cell count after excessive dose of Vinblastine. Following 40 mg. in 72 hours, W.B.C. total fell from 8000 to 200. Recovery was complete and treatment was interrupted for only 14 days.

relationship between the dose level and the degree of pain in each individual. Few patients can tolerate the pains produced by individual doses of 20 mg. Fortunately, Hodgkin's disease frequently responds to smaller weekly doses which produce only minor aches and pains, usually maximal about 36 hours after the injection which are usually ignored by the patient. Constipation is frequently referred to as a common complication of the drug but in this group of patients it has been uncommon. One patient developed severe bowel complications. Treated during the first trials of Vinblastine, he received increasing doses at weekly intervals. Fifteen mg. weekly for 2 weeks produced colicky abdominal pains which prevented him from working for 2 days after each injection: the dose was then increased to 20 mg. weekly as the blood count was satisfactory. After two injections he was admitted elsewhere as an emergency with " intestinal obstruction ". At operation the entire bowel was grossly distended but there was no obstruction. The abdominal cavity was closed with difficulty. This was obviously a form of paralytic ileus produced by the drug. The patient recovered completely.

Peripheral neuritis has been seen in two patients: recovery when the drug was stopped confirmed that Vinblastine was the cause. The clinical picture resembles

FIG. 2.—Duration of remissions induced by Vinblastine. One patient remains in remission after 69 months.

that seen with Vincristine, but in our experience it is a rare complication after Vinblastine.

*Alopecia* occurred in eight patients, but in all except one, regrowth of hair occurred in spite of continued injections. One patient regrew only sparse hair; he had already had complete hair loss following a course of cyclophosphamide 8 months previously. No dermatological complications have been attributed to Vinblastine.

*Tumour Response*

The natural fluctuations of Hodgkin's disease make it necessary to discount any improvement of less than 2 months duration. Of the 124 patients treated with Vinblastine for 1 month or longer, 31 have failed to show an objective improvement which has been maintained for at least 2 months. Ninety-three patients have responded: 55 well, and 38 moderately well. The assessment of degree of response is notoriously difficult. In this series, a good response indicates a 75%–100% tumour regression as far as it can be measured clinically, using at least three marker areas. A moderate response indicates approximately 50% tumour regression. No account has been taken of subjective improvement alone.

As indicated above, Vinblastine injections are now continued for as long as the disease continues to remain uncontrolled. Rarely the drug is stopped because of side effects: sometimes difficult veins make continuation impossible. Of the patients responding for 8 weeks or more, control was maintained for 6 months in 32 (34%), for 1 year in 17 (18%) and three patients continued to respond at 2 years (Fig. 2). The longest remission so far is 69 months. This patient was first seen 14 years ago with enlarged cervical lymph nodes. A biopsy showed Hodgkin's disease; the lymph nodes responded rapidly to radiotherapy. Five years later, mediastinal lymph node enlargement occurred, and again this responded well to radiotherapy. In 1963 he developed a high temperature, night sweating and upper abdominal pain suggesting retroperitoneal lymph node involvement. The lymphogram demonstrated enlarged lymph nodes and an inferior venacavogram showed displacement of the left kidney (Fig. 3). Vinblastine injections were given at weekly intervals and later X-rays of the abdomen confirmed the dramatic symptomatic improvement (Fig. 4). He remains in excellent health 5 years and 9 months afterwards and continues to have Vinblastine 10 mg. intravenously every 2 months. In this patient the whole tempo of the disease was slow, and in retrospect he would probably have responded just as well if the retroperitoneal nodes had been treated with radiotherapy. Nevertheless, this is a very satisfactory response and the patient continues to be delighted with his progress.

It is concluded that Vinblastine is a relatively safe and effective drug in the control of Hodgkin's disease that has advanced beyond the stage that is potentially curable by radiotherapy.

### SUMMARY

The author reports 126 patients with Stage III or IV Hodgkin's disease treated with Vinblastine sulphate for at least 4 weeks. Remission of 8 weeks or longer occurred in 93 patients (74%). Of these 32 were maintained for 6 months and 17 for 1 year. The longest remission to date is 69 months.

Complications were unusual. Serious bone marrow depression occurred only in the presence of widespread disease and after previous treatment. Thrombocytopenia was not seen in association with the more temporary fluctuations in the white count.

The author is very grateful to the many consultants who have referred patients for treatment. Part of the expense of this study was defrayed by the British Empire Cancer Campaign for Research.

### REFERENCE

BLEEHEN, N. M. AND JELLIFFE, A. M.—(1965) *Br. J. Cancer*, **19**, 268.

---

### EXPLANATION OF PLATES

FIG. 3.—Retroperitoneal node enlargement demonstrated by lymphangiogram and inferior venacavogram. The left ureter is displaced by the retroperitoneal mass.

FIG. 4.—Retroperitoneal node response to Vinblastine injections. Improvement continues for 69 months.

3

Jelliffe.

4

Jelliffe.